# Expression and role of regulator of G‐protein signaling 5 in squamous cell carcinoma of the tongue

**DOI:** 10.1002/cre2.166

**Published:** 2019-03-04

**Authors:** Yushi Abe, Sachiko Ogasawara, Jun Akiba, Yoshiki Naito, Reiichiro Kondo, Ken Nakamura, Jingo Kusukawa, Hirohisa Yano

**Affiliations:** ^1^ Department of Pathology Kurume University School of Medicine Kurume Japan; ^2^ Dental and Oral Medical Center Kurume University School of Medicine Kurume Japan; ^3^ Department of Diagnostic Pathology Kurume University Hospital Kurume Japan

**Keywords:** immunohistochemical staining, oral cancer, RGS5, tongue

## Abstract

Regulator of G‐protein signaling (RGS) 5 acts as a GTPase‐activating protein to negatively regulate G‐protein signaling. RGS5 is reportedly related to the invasion and metastasis of cancers, such as nonsmall lung cancer and hepatocellular carcinoma. We examined RGS5 expression and its relationship with invasion in squamous cell carcinoma (SCC) of the tongue. For immunohistochemical analysis of RGS5, we used SCC tissues of the tongue obtained from 43 patients. We examined the relationship between RGS5 expression in the deepest point of invasion and clinicopathological features. Because the invasion and metastasis of cancers are related to epithelial‐mesenchymal transition (EMT), we carried out staining for N‐cadherin, vimentin, and E‐cadherin to examine the relationship between EMT and RGS5. RGS5 expression in the deepest point of invasion in SCC of the tongue was observed in 32 cases (75%). Immunohistochemical analysis revealed a significant correlation between RGS5 expression in the aggressive invasion pattern, invasion depth, and lymphovascular invasion. Kaplan–Meier analysis revealed that high RGS5 expression was associated with postoperative early lymph node metastasis. Further, a significant positive correlation was observed between RGS5 and N‐cadherin (*P* = 0.0003) and vimentin (*P* < 0.0001). In contrast, E‐cadherin and RGS5 or vimentin were significantly negatively correlated (*P* < 0.0001–0.005). The findings indicate that RGS5 expression is related to tumor invasion and EMT in SCC of the tongue and that RGS5 may predict postoperative early lymph node metastasis. Therefore, RGS5 may be a useful prognostic biomarker of the surgically resected SCC and a potential target of molecular therapy for treating SCC of the tongue.

## 1 INTRODUCTION

More than 90% of oral malignancies in the head and neck region are squamous cell carcinoma (SCC). Overall, oral cancer (when oropharyngeal sites are included) is the most common cancer worldwide (Warnakulasuriya, 2009). The degree of differentiation, vascular invasion, tumor invasion distance (Almangush et al., 2015; Brandwein‐Gensler et al., 2005; Li et al., 2013; Woolgar, 2006; Woolgar & Triantafyllou, 2011), and tumor growth pattern of oral SCC (OSCC) affect prognosis. OSCC can present an expansive and/or infiltrative growth pattern. Invasion patterns of OSCC were classified as described previously by Li et al. (2013), in which the pattern of invasion was evaluated based on worst pattern of invasion (WPOI) and classified as nonaggressive (WPOI Types 1–3) and aggressive (WPOI Types 4 and 5) patterns. Types 1 and 2 show clear expansive growth pattern. On the other hand, Types 4 and 5 often show infiltrative growth pattern. Type 3 is intermediate between Types 1 and 2 and Types 4 and 5. In cases with infiltrative growth, the tumor cells show poor cell adhesiveness with frequent lymph node metastasis. To examine the effects of infiltrative growth on prognosis, previous studies have focused on the histological characteristics of this type (Li et al., 2013).

Epithelial‐mesenchymal transition (EMT) occurs when epithelial tumor cells invade the surrounding tissue, which induces the functions of various related factors. EMT reduces cellular adhesion by transforming epithelial cells into mesenchymal cells. These cells feature high transferability and invasiveness. Epithelial marker expression decreases with reduced cellular adhesion, whereas cells with high transferability and invasiveness exhibit increased expression of mesenchymal cell markers (Kalluri & Weinberg, 2009). Therefore, many associations have been detected between factors involved in invasion and factors connecting and activating EMT. Hu et al. (2013) reported that regulator of G‐protein signaling (RGS) 5 is related with tumor invasion by inducing EMT of hepatocellular carcinoma (HCC) cells. We also demonstrated that RGS5 expression is closely related with portal vein invasion and intrahepatic metastasis in HCC (Umeno et al., 2018). The RGS family is a group of multifunctional proteins that regulate cellular signaling events downstream of G‐protein coupled receptors (Hurst & Hooks, 2009). RGS5 is a member of the RGS family and acts as a GTPase‐activating protein (GAP) composed of heterotrimeric G‐protein α‐subunits that negatively regulate G‐protein signaling. RGS5 expression has been detected in the heart, lung, skeletal muscle, and small intestine and is involved in tumor angiogenesis and gestational hypertension. RGS5 is reportedly related to the invasion and metastasis of cancers, such as HCC and nonsmall lung cancer (Hu et al., 2013; Huang, Song, Wang, Han, & Chen, 2012). Involvement of Gαq and/or Gαi subunits of G protein in EMT was reported in these cancers, but their results were inconsistent (Hu et al., 2013; Huang et al., 2012). There have been no studies on the role of RGS5 in OSCC. In this study, we investigated EMT‐related factors and RGS5.

## 2 MATERIALS AND METHODS

### 2.1 Tissue samples

We selected patients with primary tongue cancer who had not undergone preoperative treatments, such as chemotherapy and/or radiotherapy, at Kurume University Hospital between 2011 and 2015 (Table 1). Resected tissues were fixed using 10% buffered formalin, sectioned at 4‐μm thickness, and the sections were stained using hematoxylin–eosin. Pathological diagnosis was performed according to the *World Health Organization Classification of Head and Neck Tumors 4th Edition* (El‐Naggar, Chan, Grandis, Takata, & Slootweg, 2017).

**Table 1:** Clinicopathological characteristics of 43 squamous cell carcinoma of the tongue cases

Age (years; mean ± *SD*)	68.8 ± 15.86
Gender (male/female)	22/21
Tumor size (mm; mean ± *SD*)	20.37 ± 9.22
T classification, *n* (%)	
T1	20 (46.5)
T2	22 (51.1)
T3	1 (2.5)
N classification, *n* (%)	
N0	40 (93.0)
N1	1 (2.3)
N2	2 (4.6)
Pattern of invasion, *n* (%)	
Expansive type	7 (16.2)
Intermediate type	22 (51.1)
Infiltrative type	14 (32.5)
Differentiation, *n* (%)	
Well differentiated	34 (79.0)
Moderately differentiated	7 (16.2)
Poorly differentiated	2 (4.6)
Depth of invasion (mm), *n* (%)	
<4	21 (48.8)
4≦	22 (51.1)
Lymphatic vessel invasion, *n* (%)	8 (18.6)
Vascular invasion, *n* (%)	8 (18.6)
Lymph node metastasis after surgery, *n* (%)	16 (37.2)
(months; mean ± *SD*)	6.76 ± 1.69

*Note*. *SD*: standard deviation.

**Figure 1:**
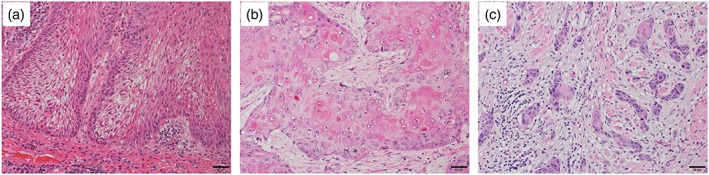
Representative photomicrographs of squamous cell carcinoma of the tongue with three different invasion patterns in the invasive portions: (a) expansive type, (b) intermediate type, and (c) infiltrative type

Invasion patterns of OSCC were classified previously by Li et al. (2013), in which the pattern of invasion was evaluated based on the WPOI and classified into the following categories: nonaggressive (WPOI Types 1–3) and aggressive (WPOI Types 4 and 5) patterns. Types 1 and 2 present pushing border and finger‐like growth, respectively, and show a clear expansive growth pattern. Types 4 and 5 present small tumor islands (<15 cells per island) and tumor satellites (≥1 mm from the main tumor or next closest satellite), respectively, and often show an infiltrative growth pattern. Type 3 presents large (>15 cells) separated islands and shows a growth pattern that is a mixture of those shown by Types 1 and 2 and Types 4 and 5. On the basis of these findings, we defined Types 1 and 2 as “expansive type” and Types 4 and 5 as “infiltrative type.” We defined Type 3 as “intermediate type.” (Figure 1) These cases feature different depth of invasion.

We defined “invasive portion” as the deepest tumor nests far from the superficial squamous cells. The “noninvasive portion” is usually located near superficial squamous cells and shows no clear evidence of invasion. The tumor cells are sometimes contiguous to squamous epithelial mucosa, resembling the features of in situ carcinoma.

We used a cutoff value of 4 mm for tumor depth of invasion. This cutoff value has been widely used in other studies. The depth of invasion of tumors was measured at the deepest point of invasion from the level of the basement membrane of the adjacent normal mucosa.

This study was approved by the ethics committee of Kurume University (approval no. 332).

### 2.2 Immunohistochemical analysis

We performed immunohistochemical analysis of paraffin‐embedded sections using RGS5, N‐cadherin, vimentin, and E‐cadherin antibodies. For RGS5, the sections were deparaffinized and rehydrated in xylene and 100% graded ethanol, respectively. The sections were subsequently soaked in Target Retrieval Solution, pH 6 (Agilent Technologies, Inc., Santa Clara, CA, USA) and treated at 110°C in a pressure cooker for 60 min. Immunohistochemical staining was performed using a CSA II system (Agilent Technologies, Inc.) according to the manufacturer's protocol. Nonspecific binding sites were blocked for endogenous peroxidase activity using a blocking solution (Agilent Technologies, Inc.) for 5 min, followed by incubation of the sections with primary antibody for 60 min at room temperature. The primary antibody was a mouse monoclonal antihuman RGS5 antibody (clone, 1C1; cat no. NBP2‐00880; dilution, 1:250; Novus Biologicals, LLC, Littleton, CO, USA). For the RGS5 profile, we evaluated cytoplasmic staining as described by Umeno et al. (2018). Normal pancreatic islets of Langerhans were used as positive control for RGS5. Nuclear RGS5 expression in the superficial part of normal squamous epithelium is considered the positive internal control. The negative control was prepared by replacing the primary anti‐RGS5 antibody with normal mouse IgG at the same concentration.

Immunohistochemistry analysis for N‐cadherin, vimentin, E‐cadherin, and p16 was performed using the BenchMark XT device (Ventana Medical Systems, Inc., Tucson, AZ, USA). Anti‐N‐cadherin antibody (clone, IAR06; dilution 1:100, Leica Biosystems Newcastle Ltd, UK), anti‐E‐cadherin antibody (clone, NCH‐38; dilution 1:100, Dako, Glostrup, Denmark), antivimentin antibody (clone, V9; dilution 1:100, Dako), anti‐p16 antibody (clone, sc‐56330; dilution 1:200, Santa Cruz Biotechnology, Inc., Dallas, TX, USA), anticytokeratin 5/6 (clone, D5/16 B4; dilution 1:100, Dako), and anti‐p16 antibody (clone, sc‐56330; dilution 1:200, Santa Cruz Biotechnology, Inc.) were used. This automated system uses the streptavidin‐biotin complex method with 3,3′‐diaminobenzidine as a chromogenic substrate (Ventana iVIEW DAB Detection Kit). We also used this system and anti‐D2‐40 antibody (clone, D2‐40; dilution 1:1, Nichirei Bioscience, Tokyo, Japan) to identify lymph vessel invasion of tumor cells. Elastica van Gieson stain was employed to identify venous invasion of tumor cells.

Tumor cells in the invasive portion of the tumor where RGS5 and vimentin were stained in the cytoplasm and where N‐cadherin and E‐cadherin were stained in the cell membrane were considered as positive. Staining intensity was evaluated using the following methods. For RGS5, staining intensity of the invasive portion was scored on a 0–3 scale compared with the noncancerous squamous epithelium as follows: 0, intensity in the invasive portion was equal to that in the noncancerous squamous epithelium; 1, intensity in the invasive portion was slightly higher than that in the noncancerous squamous epithelium; 2, moderately higher; and 3, strongly higher. Furthermore, an RGS5 score of 0 or 1 was considered as low expression, whereas an RGS5 score of 2 or 3 was considered as high expression. The staining intensity of N‐cadherin was evaluated as follows: Nonstaining was scored as 0, weak staining intensity was 1, medium staining intensity was 2, and equivalent staining intensity was 3, as compared with the staining intensity of nerve cells. The staining intensity of vimentin was evaluated as follows: Staining intensity less than that of mesenchymal cells, such as vascular endothelial cells and fibroblasts, was scored as 0, weak staining intensity was scored as 1, moderate staining intensity was scored as 2, and equivalent staining intensity was scored as 3. The staining intensity of E‐cadherin was evaluated as follows: Staining intensity less than that of noncancerous squamous epithelium was scored as 0, weak staining intensity was scored as 1, moderate staining intensity was scored as 2, and equivalent staining intensity was scored as 3. Histological and immunohistochemical analyses were conducted independently by two pathologists (Y. A. and R. K.). If the results were inconsistent, the decisions were made based on discussion and consensus. At least 1,000 tumor cells were counted to evaluate staining intensity.

### 2.3 Statistical analyses

JMP software version 12.0 was used for all statistical analyses (SAS Institute, Cary, NC, USA). The correlation between RGS5 immunoexpression and clinicopathological parameters was assessed by chi‐square test or Fisher's exact test. The overall survival rate was defined as the interval between the diagnosis and date of tumor metastasis (uncensored data) or the data from the last available clinical information (censored data). Comparison and estimation of cumulative survival rates were performed using Kaplan–Meier curves and the log–rank test. All tests were two‐sided, and a *P*‐value <0.05 was considered significant.

## 3 RESULTS

### 3.1 Clinicopathological characteristics

Detailed clinicopathological characteristics of the 43 cases of SCC of the tongue are shown in Table 1. The mean age of patients was 68.8 ± 15.9 years (range 32–91 years). Of the 43 patients, 22 were male and 21 were female. The mean tumor size was 20.4 ± 9.2 mm (range 7–45 mm). Twenty patients were classified as T1, 22 were T2, and one was T3. Forty patients were classified as N0, one was N1, and two were N2. Regarding WPOI, the expansive type, intermediate type, and infiltrative type were found in 7 (16.2%), 22 (51.1%), and 14 (32.5%) patients, respectively. Regarding histological grading, well‐differentiated, moderately differentiated, and poorly differentiated tumors were found in 34 (79.0%), 7 (16.2%), and 2 (4.6%) patients, respectively. Twenty‐one cases showed a depth of invasion <4 mm, whereas the others showed values ≥4 mm. Tumor depth of invasion was measured by the deepest point of invasion from the level of the basement membrane of the adjacent normal mucosa. Eight cases (18.6%) had lymphatic vessel invasion, and eight cases (18.6%) had vascular invasion.

There were no deaths among the 43 cases. Two cases had localized recurrence (2 and 48 months), and 16 cases (37.2%) had regional recurrence. The median time to recurrence was 6.8 ± 1.7 months (range 2–24 months).

### 3.2 Expression pattern of RGS5 in SCC of the tongue

**Figure 2:**
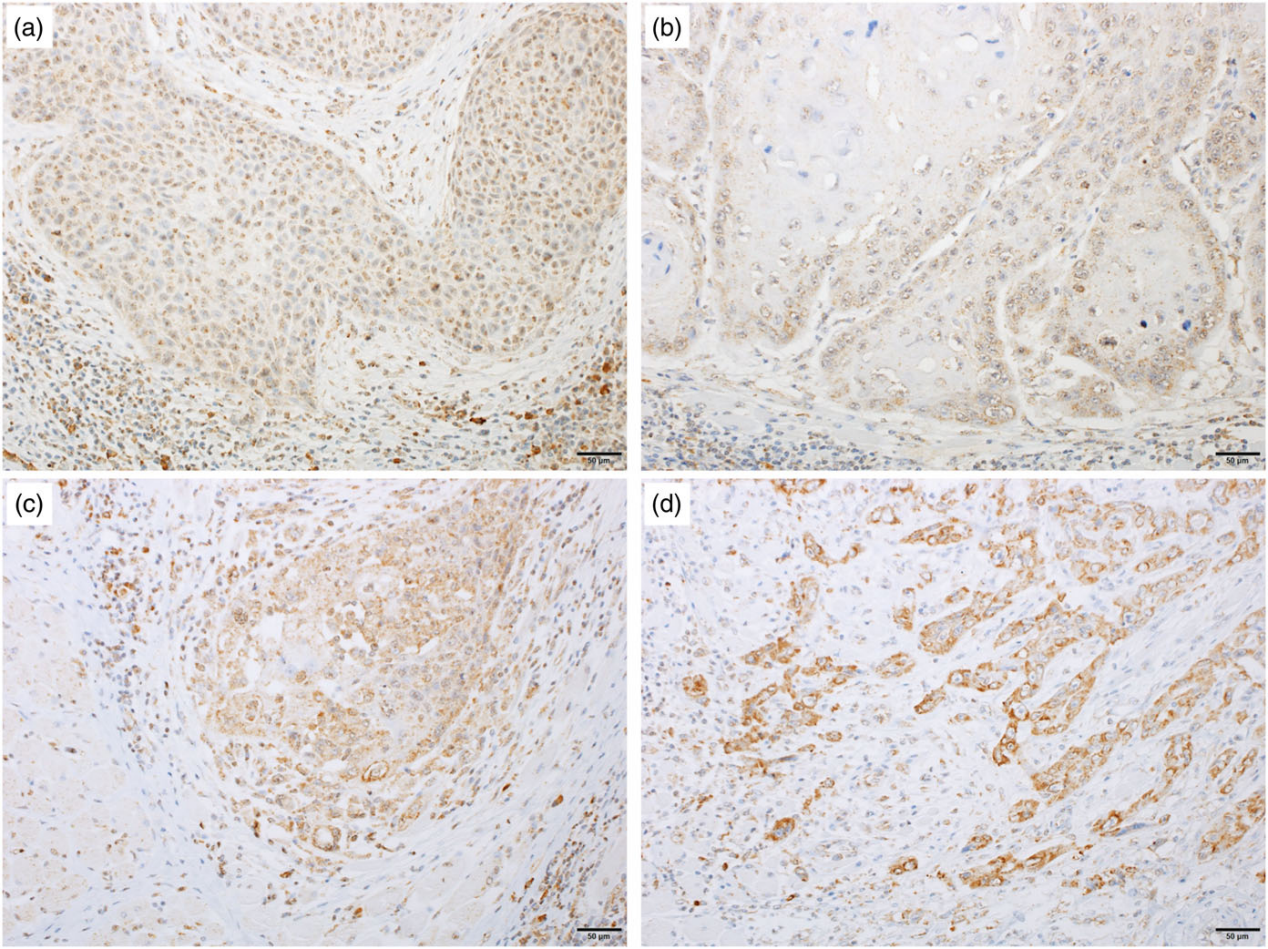
Immunostaining of regulator of G‐protein signaling 5 in squamous cell carcinoma of the tongue. The staining intensity was graded into four levels: (a) Score 0: negative; (b) Score 1: weakly positive; (c) Score 2: moderately positive; and (d) Score 3: strongly positive

**Figure 3:**
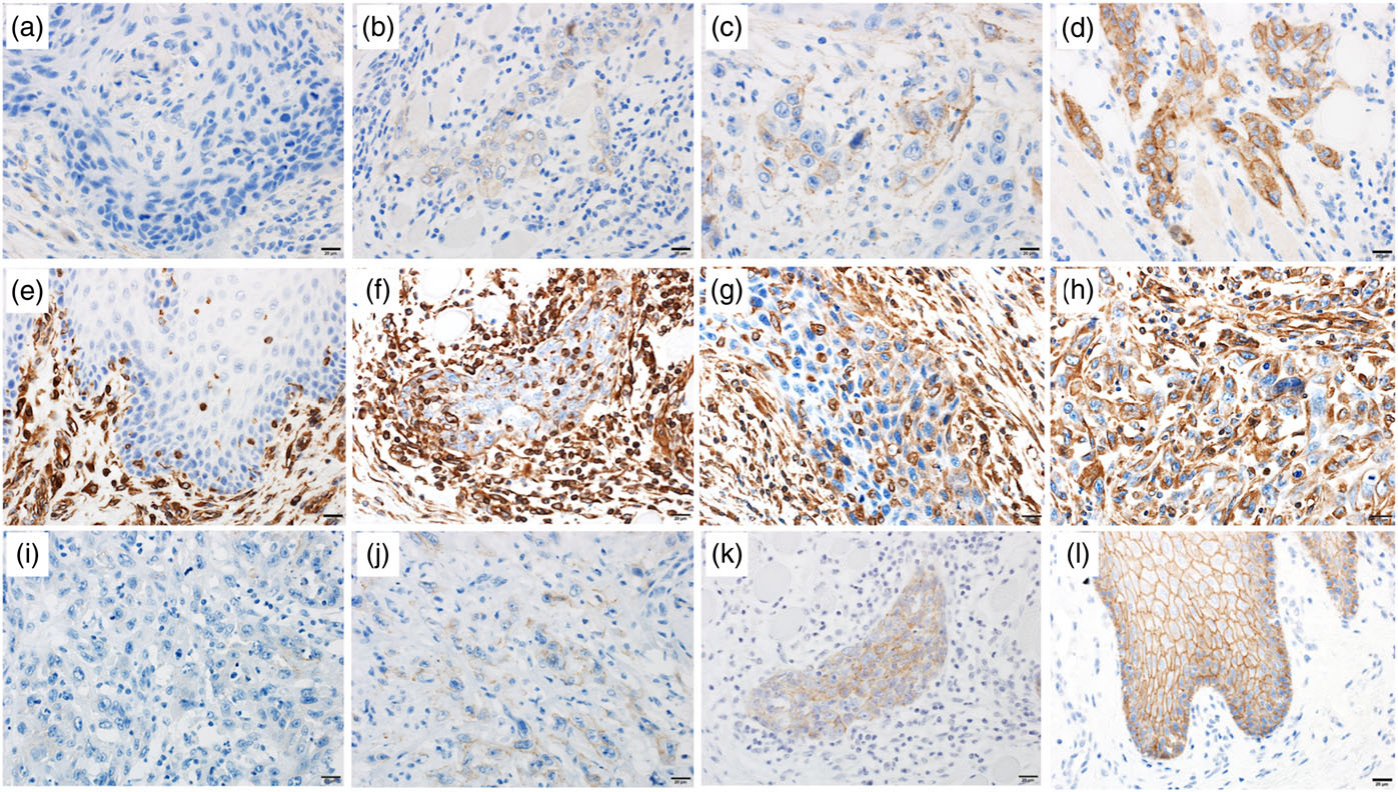
Immunostaining of N‐cadherin, vimentin, and E‐cadherin in squamous cell carcinoma of the tongue. The staining intensity of N‐cadherin was graded into four levels: (a) Score 0: negative; (b) Score 1: weakly positive; (c) Score 2: moderately positive; and (d) Score 3: strongly positive. The staining intensity of vimentin was graded into four levels: (e) Score 0: negative; (f) Score 1: weakly positive; (g) Score 2: moderately positive; and (h) Score 3: strongly positive. The staining intensity of E‐cadherin was graded into four levels: (i) Score 0: negative; (j) Score 1: weakly positive; (k) Score 2: moderately positive; and (l) Score 3: strongly positive

RGS5 expression in the normal mucosa was detected in the superficial part of the tumor in the cell nucleus, whereas RGS5 expression in the invasive portion was observed in the cytoplasm, demonstrating the localized expression of RGS5 changes with tumor invasion. Dysplastic cells observed in the vicinity of SCC in some cases also expressed nuclear RGS5. We compared and quantitated the RGS5 expression between normal squamous epithelium and SCC. RGS5 expression in the invasive portion of SCC of the tongue was observed in 32 cases (75%). Representative microphotographs of RGS5 are shown in Figure 2. Inter‐rater correlation was obtained by fitting the mixed‐effect model. Estimated inter‐rater correlation was 0.92 indicating excellent agreement between the two raters (Y. A. and R. K.). Representative microphotographs of N‐cadherin, vimentin, and E‐cadherin are shown in Figure 3.

The number of cases with high RGS5 expression was significantly higher for the infiltrative type and that with low RGS5 expression was significantly lower for the expansive type (Table 2; *P* = 0.0037). In terms of invasion distance, a significantly larger number of high expression cases showed a depth of invasion ≥4 mm compared with low expression cases (Table 2; *P* = 0.0463). Additionally, seven of the eight cases with lymphatic invasion showed significantly high expression compared with the low expression cases (Table 2; *P* = 0.0238). We performed immunostaining of p16 protein, which is a marker of human papillomavirus infection, for 43 cases. Seven and 16 cases with high RGS5 expression were positive and negative, respectively, for p16 expression, whereas three and 17 cases with low RGS5 expression were positive and negative, respectively, for p16 expression. No significant difference was observed in p16 expression between high and low RGS5 expression cases (Table 2; *P* = 0.2321).

**Table 2:** The association between RGS5 and various clinicopathologic factors

RGS5 expression			
Characteristics	High expression	Low expression	*P* value
No. of patients	20	23	
Age (years)			0.6059
≦70	11	8	
70<	12	12	
Gender			0.6386
Male	11	11	
Female	12	9	
Tumor size			0.9202
<20	13	9	
20≦	10	11	
T classification			0.4373
T1	10	11	
T2	12	9	
T3	1	0	
N classification			0.6313
N0	21	19	
N1 + N2	2	1	
Pattern of invasion			0.0037
Expansive type	1	6	
Intermediate type	10	12	
Infiltrative type	12	2	
Differentiation			0.1667
Well	17	18	
Moderate + Poor	6	2	
Depth of invasion (mm)			0.0463
<4	8	13	
4≦	15	7	
Lymphatic vessel invasion			0.0238
Absent	16	19	
Present	7	1	
Vascular invasion			0.5689
Absent	18	17	
Present	5	3	
Lymph node metastasis after surgery			0.1684
Absent	9	4	
Present	14	16	
p16			0.2321
Absent	16	17	
Present	7	3	

*Note*. RGS5: regulator of G‐protein signaling 5; Well: well‐differentiated squamous cell carcinoma; Moderate: moderately differentiated squamous cell carcinoma; Poor: poorly differentiated squamous cell carcinoma.

**Figure 4:**
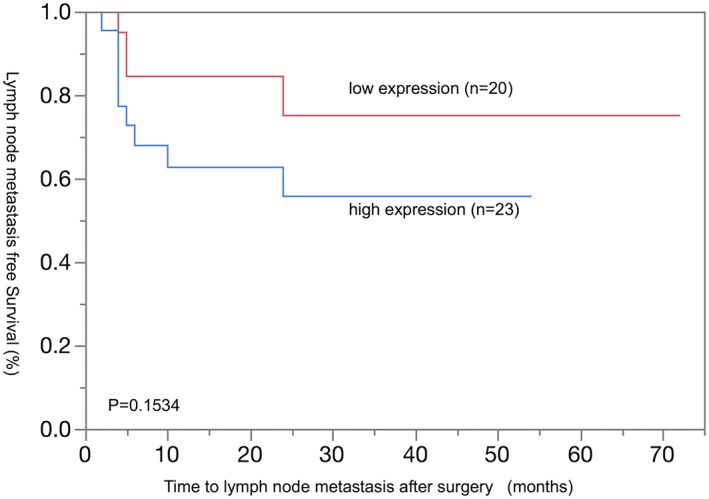
Relationship between expression of regulator of G‐protein signaling 5 and time to lymph node metastasis after surgery

Although there was no significant difference in lymph node metastasis curve between high and low RGS5 expression groups (Figure 4; *P* = 0.1534), recurrence occurred in all cases by 25 months after surgery. Lymph node metastasis after surgery means removed when neck failed subsequent to surgery. In addition, lymph node metastasis was observed in 13 of 14 cases (92%) in the high expression group by 10 months after the operation. Patients with N0 were followed up for 3 to 7 years.

### 3.3 Relationship between WPOI and expression of RGS5, N‐cadherin, vimentin, and E‐cadherin

**Figure 5:**
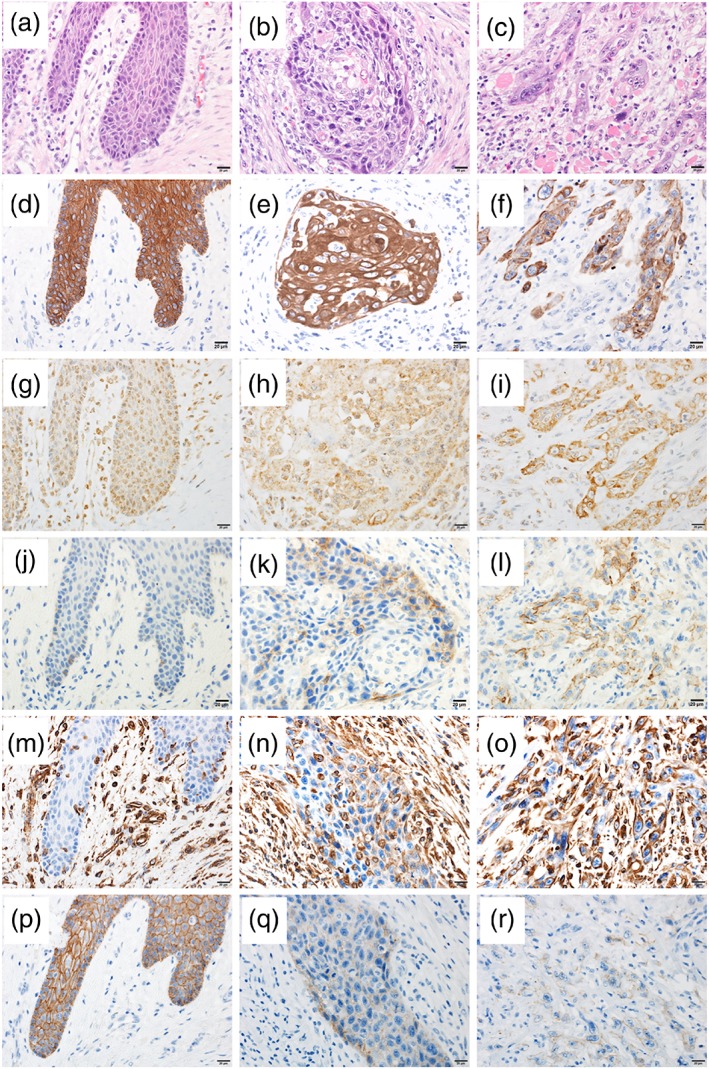
Representative photomicrographs of hematoxylin and eosin staining and staining for CK5/6, regulator of G‐protein signaling 5, N‐cadherin, vimentin, and E‐cadherin in squamous cell carcinoma of the tongue with different invasion patterns. (a), (b), and (c) show hematoxylin and eosin in the expansive, intermediate, and infiltrative types, respectively. (d), (e), and (f) show CK5/6 expression in the expansive, intermediate, and infiltrative types, respectively. (g), (h), and (i) show regulator of G‐protein signaling 5 expression in the expansive, intermediate, and infiltrative types, respectively. (j), (k), and (l) show N‐cadherin expression in the expansive, intermediate, and infiltrative types, respectively. (m), (n), and (o) show vimentin expression in the expansive, intermediate, and infiltrative types, respectively. (p), (q), and (r) show E‐cadherin expression in the expansive, intermediate, and infiltrative types, respectively

E‐cadherin was strongly expressed in the invasive portion of the expansive type (Figure 5p), whereas there was low expression of RGS5, N‐cadherin, and vimentin (Figure 5g,j,m). Moderate expression of RGS5, N‐cadherin, vimentin, and N‐cadherin was observed in the invasive portion of intermediate type (Figure 5h,k,n,q). Low expression of E‐cadherin was observed in the invasive portion of infiltrative type (Figure 5r), whereas RGS5, N‐cadherin, and vimentin were strongly expressed (Figure 5i,l,o). The expressions of RGS5 and vimentin were significantly higher in the infiltrative type than in the expansive and intermediate types (*P* < 0.05 and *P* < 0.01, respectively). E‐cadherin expression was significantly higher in the expansive type than in the infiltrative type (*P* < 0.01; Table 3).

**Table 3:** Relationships between invasion patterns and immunohistochemical expression of RGS5, vimentin, or E‐cadherin

Number of positive cases			
	RGS5	Vimentin	E‐cadherin
Expansive type (*n* = 7) Intermediate type (*n* = 22) Infiltrative type (*n* = 14)	$\left.\begin{array}{l} \left.\begin{array}{l} 1\;\left(14\%\right)\\ 10\;\left(45\%\right) \end{array}\right]*\\ 12\;\left(85\%\right) \end{array}\right]*$	$\left.\begin{array}{l} \left.\begin{array}{l} 2\;\left(28\%\right)\\ 12\;\left(54\%\right) \end{array}\right]*\\ 13\;\left(92\%\right) \end{array}\right]**$	$\left.\begin{array}{l} 7\;\left(100\%\right)\\ 17\;\left(77\%\right)\\ 7\;\left(50\%\right) \end{array}\right]*$

*Note*. RGS5: regulator of G‐protein signaling 5.

* *P* < 0.05.

** *P* < 0.01.

**Figure 6:**
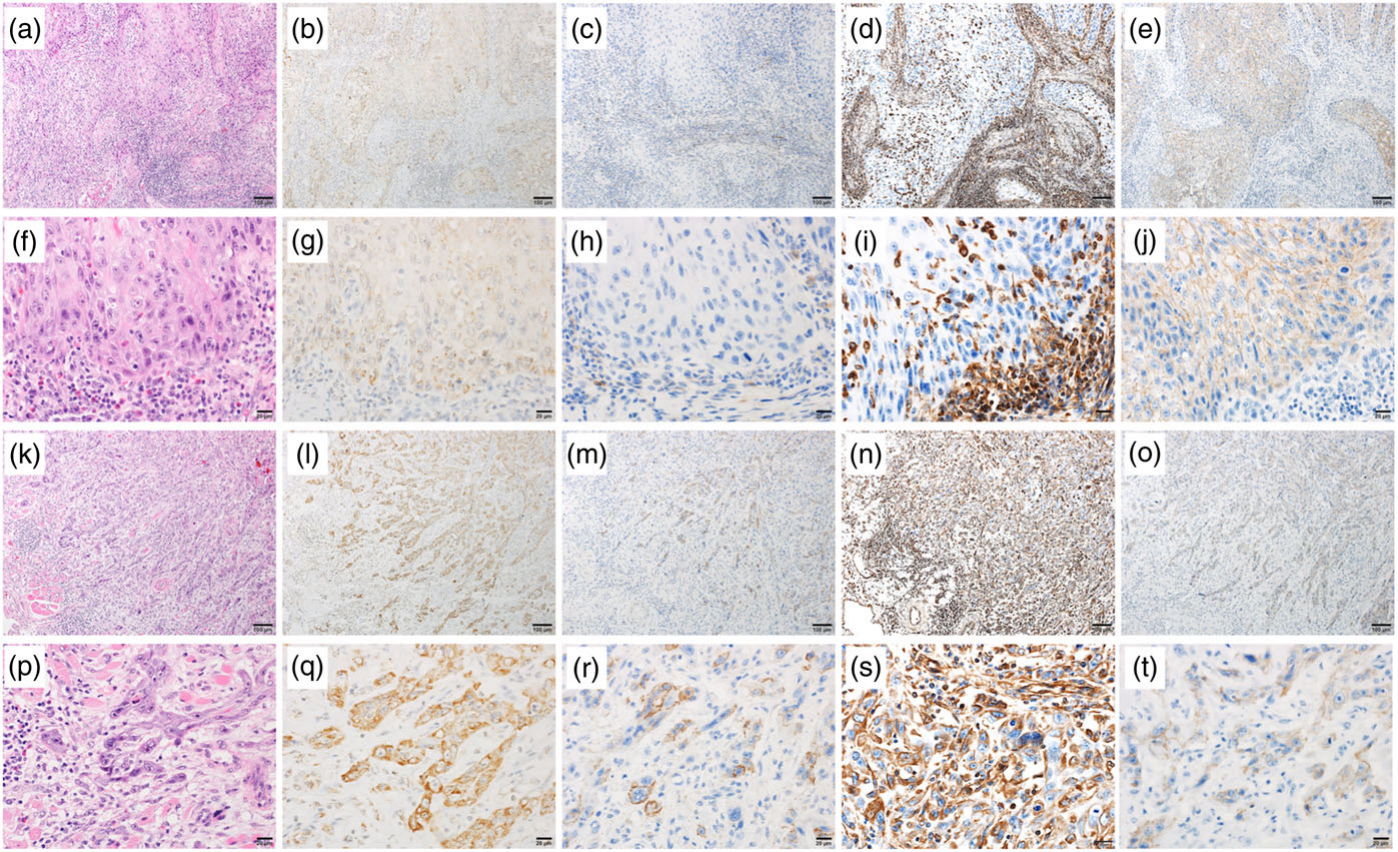
Representative photomicrographs of hematoxylin and eosin (H&E), CK5/6, regulator of G‐protein signaling (RGS) 5, N‐cadherin, vimentin, and E‐cadherin in squamous cell carcinoma of the tongue in noninvasive and invasive portions. (a), (b), (c), (d), and (e) show H&E, RGS5, N‐cadherin, vimentin, and E‐cadherin in noninvasive portions at 10× magnification. (f), (g), (h), (i), and (j) show H&E, RGS5, N‐cadherin, vimentin, and E‐cadherin in noninvasive portions at 40× magnification. (k), (l), (m), (n), and (o) show H&E, RGS5, N‐cadherin, vimentin, and E‐cadherin in invasive portions at 10× magnification. (p), (q), (r), (s), and (t) show H&E, RGS5, N‐cadherin, vimentin, and E‐cadherin in invasive portions at 40× magnification

Photomicrographs of hematoxylin and eosin staining and examinations of RGS5, N‐cadherin, vimentin, and E‐cadherin in noninvasive and invasive portions of infiltrative type are presented in Figure 6. SCC cells in the noninvasive portion of the infiltrative type showed nuclear RGS5 expression (Figure 6b,g) and moderate cell membrane E‐cadherin expression (Figure 6e,j). There was no expression of N‐cadherin and vimentin in the noninvasive portion (Figure 6c,d,h,i). Low expression of E‐cadherin was observed in the invasive portion of infiltrative type (Figure 6o,t), whereas RGS5 and vimentin showed strong expression (Figure 6l,n,q,s), and N‐cadherin showed moderate expression (Figure 6m,r).

### 3.4 Correlation between RGS5 expression and expressions of N‐cadherin, vimentin, and E‐cadherin

**Figure 7:**
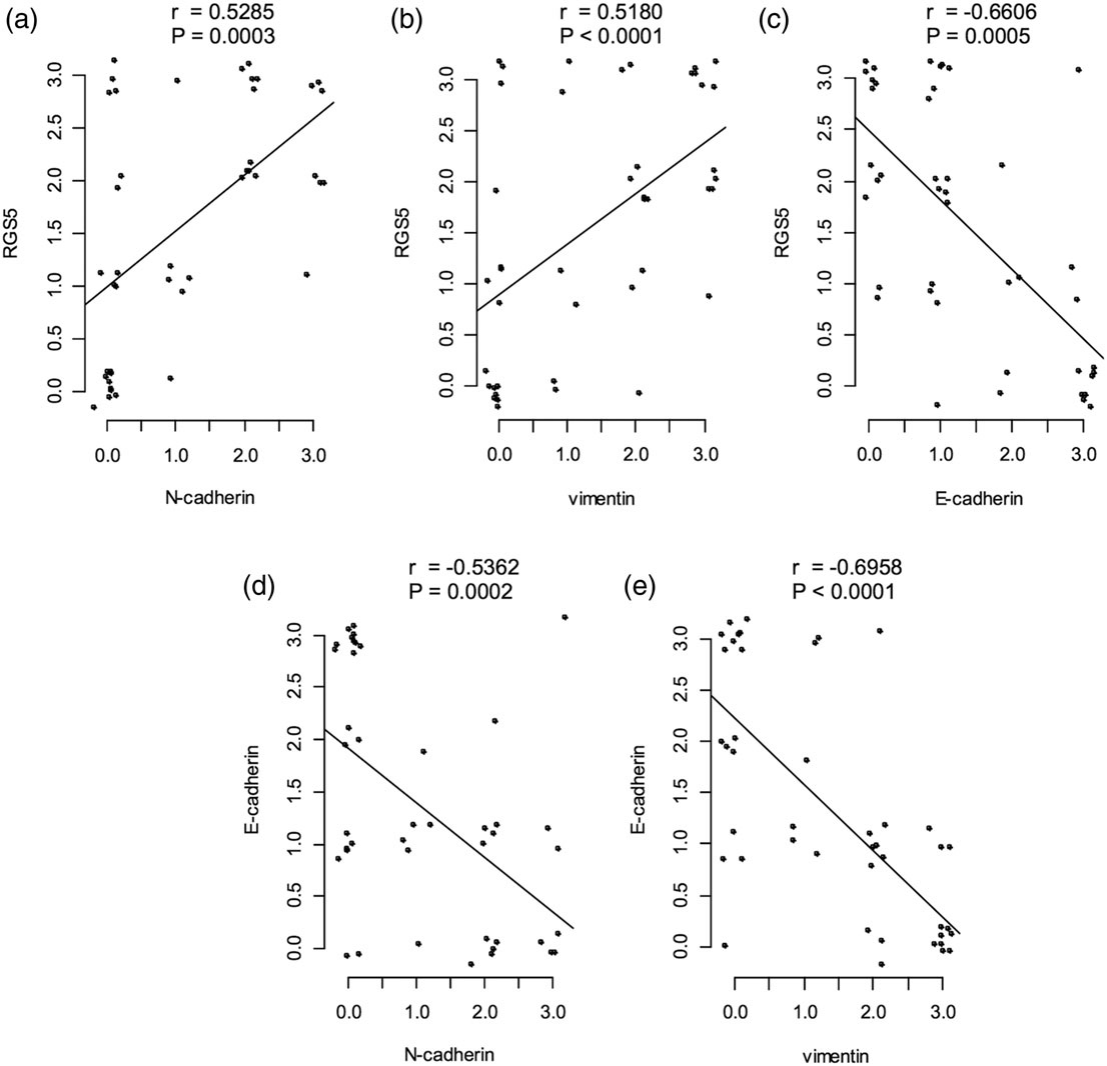
Correlation among regulator of G‐protein signaling (RGS) 5, N‐cadherin, vimentin, and E‐cadherin expressions. (a), (b), (c), (d), and (e) show the expression relationships between RGS5 and N‐cadherin, RGS5 and vimentin, RGS5 and E‐cadherin, E‐cadherin and N‐cadherin, and E‐cadherin and vimentin, respectively. There was a significant positive correlation between RGS5 and N‐cadherin (a) and vimentin (b). In contrast, there was a significant negative correlation between E‐cadherin and RGS5 (c), N‐cadherin (d), and vimentin (e)

A negative correlation was detected between RGS5 and E‐cadherin, between N‐cadherin and E‐cadherin, and between vimentin and E‐cadherin (*P* = 0.0005, 0.0002, and <0.0001, respectively). A positive correlation was evident between RGS5 and N‐cadherin and between RGS5 and vimentin (*P* = 0.0003 and <0.001, respectively; Figure 7).

## 4 DISCUSSION

In this study, we found that RGS5 appears to be involved in the aggressive biological features of OSCC, which can be mediated by EMT of SCC of the tongue. Several studies have examined the relationship between RGS5 and carcinoma. In renal cell carcinoma, strong expression of RGS5 was observed in the vascular endothelium of the tumor stromal area compared with the expression in the vascular of the normal kidneys. In gastric carcinoma and nonsmall cell lung carcinoma, RGS5 expression was positively correlated with tumor differentiation, and low RGS5 expression was associated with aggressive properties (Huang et al., 2012; Wang et al., 2010). In contrast, high RGS5 expression in HCC was reported to be associated with various aggressive properties, such as vascular invasion, intrahepatic metastasis, and EMT (Hu et al., 2013; Umeno et al., 2018). These reports indicate that the role of RGS5 in carcinoma is organ specific.

RGS5 is a member of the RGS family and acts as a GAP composed of heterotrimeric G‐protein α‐subunits, which negatively regulate G‐protein signaling. Heterotrimeric G‐protein α‐subunits are classified into four families based on homology and effector interactions: Gαi, Gαs, Gαq, and Gα12. RGS5 acts as GAPs for Gαi and Gαq subunits of G protein. Yao et al. (2012) demonstrated that Gαi‐1 of Gαi subunits, which is downregulated in HCC, inhibit the migration and metastasis of HCC cells. Because RGS5 can inactivate Gαi‐1, the results of Yao et al. suggest that RGS5 may promote the migration and metastasis of HCC cells. However, in nonsmall lung cancer, the opposite results were reported by Huang et al. (2012) who speculated that activation of Gαq and Gαi subunits of G protein could elicit the activation of Ras‐mitogen‐activated protein kinase pathway and the enhanced expression of EMT‐related transcription factors, such as Snail and Slug. RGS5 could inhibit the activation of Gαq and Gαi subunits of G protein and then hinder the EMT of cancer cells. The detailed molecular mechanisms of EMT are not clear in the present study. However, because high RGS expression was associated with tumor invasion in HCC and SCC of the tongue, we speculated that EMT of SCC of the tongue may be mediated by the mechanisms similar to HCC.

In this study, we found that RGS5 expression in the invasive portion of tongue cancer was significantly higher in infiltrative type than in expansive type. Given that RGS5 expression is high in the infiltrative type, RGS5 expression may reflect tumor invasiveness and infiltration patterns. Additionally, high expression of RGS5 is associated with significantly higher rates of lymphatic invasion compared with low expression, suggesting that RGS5 is related to lymphatic invasion.

Several studies revealed that the tumor invasion pattern is very important for predicting patient outcomes. Locoregional recurrence occurs at an earlier stage in aggressive invasion patterns than in nonaggressive invasion, decreasing disease‐specific survival (Li et al., 2013). Furthermore, lymph node metastasis is more common in the aggressive invasion pattern (Chang et al., 2010; Khwaja, Tayaar, Acharya, Bhushan, & Muddapur, 2018; Tanaka, Odajima, Ogi, Ikeda, & Satoh, 2003). In this study, we assessed the invasion patterns as described by Li et al. (2013). The invasion patterns are classified as expansive, intermediate, and infiltrative. The expansive type features a border pushing front, finger‐like pushing fronts, or large separated islands and is typically observed in nonaggressive tumors. The intermediate type exhibits small groups or cords of infiltrating cells. The infiltrative type may undergo marked and widespread cellular dissociation into small groups of cells or single cells and is typically observed in aggressive tumors. In this study, the infiltrative type corresponded to an aggressive invasion pattern, whereas the expansive type corresponded to a nonaggressive invasion pattern.

Generally, EMT is closely associated with the invasive growth and lymphovascular invasion of cancer. We investigated the expression of the typical epithelial cell adhesion molecule N‐cadherin and E‐cadherin and mesenchymal marker vimentin to determine whether EMT occurs during tumor growth and compared the results with RGS5 expression at the same sites. In case of the expansive growth pattern, the expression of RGS5, N‐cadherin, and vimentin in invasive portions was low but that of E‐cadherin was high. In the infiltrative growth pattern, the opposite results were obtained. These results suggest that RGS5 expression is positively correlated with N‐cadherin and vimentin expression but negatively correlated with E‐cadherin expression, which showed high expression of RGS5, suggesting the occurrence of EMT during tumor growth.

In this study, we found that RGS5 is closely associated with tumor invasion patterns, tumor invasion depth, and lymphatic invasion. In terms of RGS5 expression and postoperative lymph node metastasis, early postoperative lymph node metastasis tended to occur in the RGS5 high expression group, but the lack of a significant difference may be related to the small number of cases (*n* = 43) and short postoperative observation period. Therefore, RGS5 may be a useful prognostic biomarker of the surgically resected SCC and a potential target of molecular therapy for treating SCC of the tongue.

## CONFLICTS OF INTEREST

The authors have no conflict of interest.
